# Supplementary material for: Association between screen time and obesity in US adolescents: A cross-sectional analysis using National Survey of Children’s Health 2016–2017
Source: PLoS One. 2022 Dec 1;17(12):e0278490. doi: 10.1371/journal.pone.0278490 (PMC9714705; doi:10.1371/journal.pone.0278490)
Supplement: S1 Table — (DOCX) [file pone.0278490.s001.docx]

**S1 Table. Results of logistic regression analysis of the effect modification by sleep duration on the association between time spent watching TV or playing video games and overweight**

|  | Overweight or obese^ vs. Normal BMI | |
| --- | --- | --- |
| Sleep duration | **Hours spent watching TV or playing video games per day** | **Fully adjusted OR (95% CI)*** |
| Equal or greater than recommended hours | **None or less than 1 hour per day** | **1.00** |
| Equal or greater than recommended hours | **1-3 hours per day** | **1.36 (1.12, 1.65)** |
| Equal or greater than recommended hours | **4 hours or more per day** | **1.99 (1.48, 2.66)** |
| Fewer than the recommended hours | **None or less than 1 hour per day** | 1.08 (0.81, 1.45) |
| Fewer than the recommended hours | **1-3 hours per day** | **1.56 (1.26, 1.94)** |
| Fewer than the recommended hours | **4 hours or more per day** | **2.27 (1.60, 3.23)** |
|  |  |  |
|  | **Males** | |
| Sleep duration | **Hours spent watching TV or playing video games per day** | **Fully adjusted OR (95% CI)*** |
| Equal or greater than recommended hours | **None or less than 1 hour per day** | **1.00** |
| Equal or greater than recommended hours | **1-3 hours per day** | 1.16 (0.88, 1.54) |
| Equal or greater than recommended hours | **4 hours or more per day** | **1.61 (1.09, 2.38)** |
| Fewer than the recommended hours | **None or less than 1 hour per day** | 0.90 (0.54, 1.50) |
| Fewer than the recommended hours | **1-3 hours per day** | **1.50 (1.08, 2.07)** |
| Fewer than the recommended hours | **4 hours or more per day** | **1.85 (1.21, 2.83)** |
|  |  |  |
|  | **Females** | |
| Sleep duration | **Hours spent watching TV or playing video games per day** | **Fully adjusted OR (95% CI)*** |
| Equal or greater than recommended hours | **None or less than 1 hour per day** | **1.00** |
| Equal or greater than recommended hours | **1-3 hours per day** | **1.56 (1.19, 2.04)** |
| Equal or greater than recommended hours | **4 hours or more per day** | **2.50 (1.60, 3.90)** |
| Fewer than the recommended hours | **None or less than 1 hour per day** | 1.28 (0.90, 1.82) |
| Fewer than the recommended hours | **1-3 hours per day** | **1.62 (1.21, 2.16)** |
| Fewer than the recommended hours | **4 hours or more per day** | **2.90 (1.69, 4.99)** |

*Adjusted for age, sex, race, health status, family income, depression, anxiety, ADHD, and amount of sleep and physical activity

^ Overweight or Obese: BMI at or above the 85th percentile for age and gender
