# Supplementary material for: Association between screen time and obesity in US adolescents: A cross-sectional analysis using National Survey of Children’s Health 2016–2017
Source: PLoS One. 2022 Dec 1;17(12):e0278490. doi: 10.1371/journal.pone.0278490 (PMC9714705; doi:10.1371/journal.pone.0278490)
Supplement: Attachment — Submitted filename: Response to Reviewers.docx [file pone.0278490.s003.docx]

**PONE-D-22-15444**

**Association between screen time and obesity in US adolescents: A cross-sectional analysis using National Survey of Children’s Health 2016-2017**

**PLOS ONE**

We appreciate the thorough review and helpful suggestions by the editor and reviewers. We believe that we have addressed the reviewer comments and made the suggested changes to the manuscript. The revised manuscript is attached, and our responses to the reviewer comments are below.

**Reviewers' comments:**

1. in the introduction, you don't tell the reader what screen-time is (line 64) or what the recommendations are that you are referring to (line 67).

**Response:** **We have added a definition of screen time (line 58) and details regarding the recommended screen time per day for adolescents (line 62)**

2. What are some reasons for these differences (line 99)?

**Response: The studies that found no association between computer use and BMI were conducted in different countries (Germany, Finland, Brazil), while the study that found an association was conducted in the US. A clarification was added to the manuscript (lines 86-88)**

3. Line 133-134, were these categories response options or did you create them? I wondered why you chose to categorise.

**Response: The two screen time questions in the NSCH had response options of none, less than one hour, one hour, 2-3 hours, 4 hours or more. We combined the first two categories into: None or less than 1 hour, and the middle two categories into 1-3 hours, to preserve sample size, especially when stratifying by sex and physical activity/sleep, and to facilitate presentation of results (including interaction) and interpretation of findings. Clarification was added on line 120.**
